# The cell wall regulates dynamics and size of plasma-membrane nanodomains in *Arabidopsis*

**DOI:** 10.1073/pnas.1819077116

**Published:** 2019-06-10

**Authors:** J. F. McKenna, D. J. Rolfe, S. E. D. Webb, A. F. Tolmie, S. W. Botchway, M. L. Martin-Fernandez, C. Hawes, J. Runions

**Affiliations:** ^a^Department of Biological and Medical Sciences, Oxford Brookes University, Oxford OX3 0BP, United Kingdom;; ^b^Central Laser Facility, Research Complex at Harwell, Science and Technology Facilities Council, Rutherford Appleton Laboratory, Oxfordshire OX11 0QX, United Kingdom

**Keywords:** nanodomain, cell wall, *Arabidopsis*, cytoskeleton, single particle tracking

## Abstract

Plant plasma-membrane (PM) proteins are involved in several vital processes, such as detection of pathogens, solute transport, and cellular signaling. For these proteins to function effectively there needs to be structure within the PM allowing, for example, proteins in the same signaling cascade to be spatially organized. Here we demonstrate that several proteins with divergent functions are located in clusters of differing size in the membrane using subdiffraction-limited Airyscan confocal microscopy. Single particle tracking reveals that these proteins move at different rates within the membrane. Actin and microtubule cytoskeletons appear to significantly regulate the mobility of one of these proteins (the pathogen receptor FLS2) and we further demonstrate that the cell wall is critical for the regulation of cluster size by quantifying single particle dynamics of proteins with key roles in morphogenesis (PIN3) and pathogen perception (FLS2). We propose a model in which the cell wall and cytoskeleton are pivotal for regulation of protein cluster size and dynamics, thereby contributing to the formation and functionality of membrane nanodomains.

## Significance

The plant plasma membrane acts as the front line for cellular perception of the environment. As such, signaling and transport proteins which perceive or transport environmental signals, developmental cues, and nutrients are located within it. A number of studies have revealed that proteins located within the plasma membrane do not simply freely diffuse within its plane. Rather, proteins are localized in nanodomains. In addition to the plasma membrane, plant cells also have an extracellular matrix, the cell wall. Here we have shown that the cell wall has a role in regulating the dynamics and size of plasma-membrane nanodomains for proteins involved in morphogenesis (PIN3) and pathogen perception (FLS2).

The plasma membrane (PM) plays key roles in compartmentalization and protection of cells from the environment (1). In plants, proteins located within the PM are critical for signal perception, transduction, and the controlled import and export of molecules (2). The PM was described by the fluid mosaic model as a diffuse mixture of proteins in motion (3). However, this does not fit observations of protein spatial heterogeneity in membranes and subsequent models have been developed (4) which incorporate ordered (nano)domains, cytoskeleton corralling, and extracellular matrices as mechanisms of spatial constraint (5).

A number of proteins are known to locate to specific domains in the plant PM. The best studied of these in plants is the REMORIN family (6–8), members of which are localized in nonoverlapping PM nanodomains (6). We define nanodomains here as distinguishable submicron protein or lipid assemblies which are 20 nm to 1 µm in size (8). Only recently has a molecular function of REMORINs been demonstrated. In rice, OsREM4.1 is up-regulated by abscisic acid and interacts with OsSERK1 to down-regulate brassinosteroid signaling (9), and *Medicago* SYMREM1 is a key protein involved in segregating the receptor LYK3 into stable nanodomains during host cell infection (10). Proteins critical for normal morphogenesis and development such as PIN1 and PIN2 are localized to defined domains in the PM. PIN2 has been shown by stimulated emission depletion (STED) superresolution imaging to form clusters in the PM, with controlled endo- and exocytosis from adjacent membrane regions to the localization domain (11). Additionally, the pathogen receptor FLS2 has been shown to localize to nanodomains in the plasma membrane (12). Spatial organization of proteins in the PM is, therefore, important for development and response to the environment, but how is membrane domain patterning regulated?

The cytoskeleton and cell wall can be thought of as a continuum with the PM (2, 13). Examples of organellar interactions within this continuum include: (*i*) the microtubule-guided CesA complex determines patterns of cellulose microfibril deposition (14); (*ii*) microtubule-associated MIDD1 is involved in secondary cell wall pit formation (15); (*iii*) the CASP family of proteins forms a PM nanodomain which defines the site of Casparian strip formation (16); and (*iv*) FORMIN1 is anchored within the cell wall, spans the PM, and nucleates actin filaments as part of a mechanism for actin cytoskeleton organization (17). Actin and microtubule cytoskeletons have been shown to regulate dynamics and structure of a number of protein nanodomains in the plasma membrane (10, 18, 19). Previous work provides further evidence of this continuum as the cell wall regulates the lateral diffusion of two “minimal” PM proteins which have GFP projecting into the cell wall space (5). Minimal membrane proteins are artificially created peptides which localize to the plasma membrane. The plant cell wall is also required for normal localization of PIN2 in the membrane and hence regulation of cell polarity (20). These examples highlight the possibility that the components of the cytoskeleton/PM/cell wall continuum can regulate each other (5, 21).

A systematic study of a number of PM proteins in plant cells has demonstrated a difference in their lateral mobility (5). This was achieved by fluorescence recovery after photobleaching (FRAP) using high temporal but low spatial resolution. Subdiffraction-limited microscopy techniques have been developed over recent years and we have used Airyscan imaging (22, 23) of flat membrane sheets in *Arabidopsis thaliana* hypocotyl cells to image PM structure with high spatial resolution. We chose to use Airyscan imaging and total internal reflection fluorescence-single particle (TIRF-SP) imaging as they do not involve the use of special fluorophores required for photo-activated localization microscopy (PALM) or a high power depletion laser used in STED which causes damage of aerial tissue in plants due to the presence of light absorbing chloroplasts. By using both TIRF-SP and Airyscan we can perform fast temporal acquisition and subdiffraction-limited imaging (down to 140 nm) in all plant tissues with the use of any existing fluorophore (22).

We show that FLS2, PIN3, BRI1, and PIP2A, form clusters of differing size from 164 to 231 nm. Our investigation indicates that actin and microtubule cytoskeletons regulate the diffusion rate of the pathogen receptor FLS2 but not the hormone transporter PIN3. Furthermore, cluster size and diffusion rate of both FLS2 and PIN3 are regulated by cellulose and pectin components of the cell wall.

We hypothesize that the constraint of the cell wall on PM proteins and differential regulation by the actin and microtubule cytoskeletons can contribute to PM organization by altering protein dynamics and hence nanodomain size.

## Results

### Plasma-Membrane Proteins Form Clusters Within the Membrane.

**Fig. 1.**
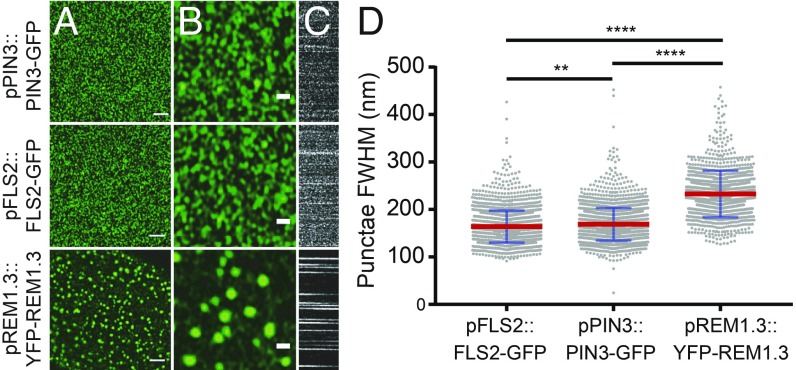
PM proteins form clusters in the hypocotyl membrane. (*A*) Airyscan imaging of pFLS2::FLS2-GFP, pPIN3::PIN3-GFP, and pREM1.3::YFP-REM1.3 clusters in the membrane of stably transformed *A. thaliana* (Scale bar, 2 µm). (*B*) Digitally magnified image of *A* showing clusters in more detail (Scale bar, 500 nm). (*C*) Kymographs showing dynamics of each nanocluster in *A* over time where x = time, y = line profile. (*D*) Scatter dot plot of FWHM measurement of cluster diameter for PM proteins in *A*. Nanodomain diameter differs significantly for each protein pair. Red lines show mean value, blue error bars show SD. **P < 0.01 and ****P < 0.0001, ANOVA with multiple comparisons.

Several well-characterized PM proteins which have a variety of functions were studied to determine how different proteins are organized in the PM and whether their dynamic behaviors differ. Determination of nanodomain full width half maximum (FWHM) demonstrated that proteins form clusters within the PM which are not resolved by diffraction-limited confocal imaging (Fig. 1 and *SI Appendix*, Fig. S1). With fluorochrome imaging, FWHM is a measure that relates to apparent domain size and visualized clusters characterize the nanodomains observed. Protein clusters were observed and measured for the auxin transporter PIN3 (puncta FWHM = 166.7 ± 31.1 nm), the pathogen receptor FLS2 (puncta FWHM = 164.3 ± 32.0 nm), the hormone receptor BRI1 (puncta FWHM = 172.6 ± 41.3 nm), and the aquaporin PIP2A (puncta FWHM = 194.3 ± 66.8 nm) (Fig. 1 and *SI Appendix*, Fig. S1). Cluster diameter was determined by FWHM measurements of line profiles over randomly selected nanodomains. Each protein observed had a nanodomain diameter below the theoretical 250-nm resolution limit of confocal microscopy (Fig. 1*D*) (24). Compared with REM1.3 (puncta FWHM = 231.0 ± 44.8 nm, Fig. 1) which is known to form highly stable nanodomains resolvable by confocal microscopy within the PM of hypocotyl cells (6), FLS2 and PIN3 clusters are significantly smaller and more dynamic within the membrane (Fig. 1*C* and *SI Appendix*, Fig. S1).

### Proteins Move at Different Speeds Within the Membrane.

**Fig. 2.**
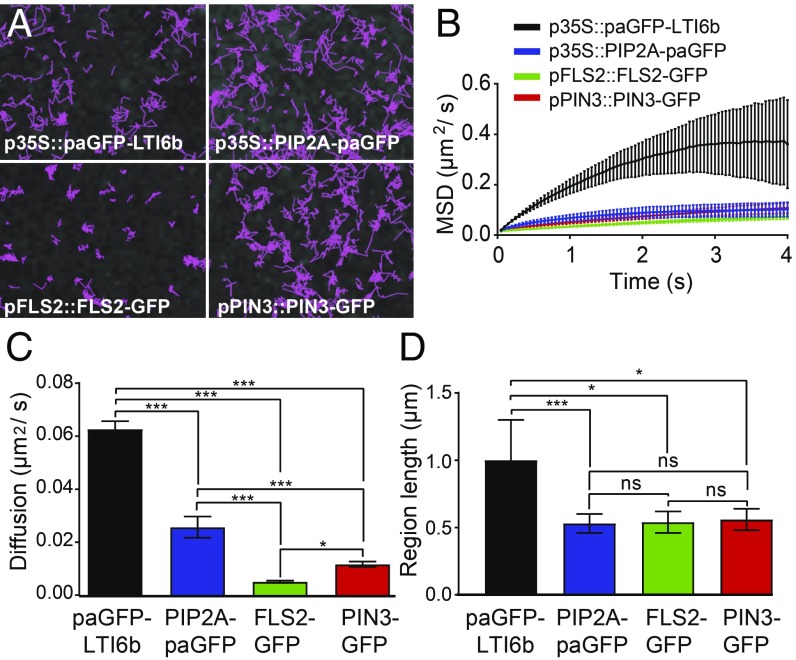
TIRF-SPT imaging of PM proteins. (*A*) TIRF-SPT of PM proteins in the hypocotyl membrane. Images show tracks followed by single labeled particles over 60 s. Some proteins, e.g., FLS2-GFP, are much more constrained in their lateral mobility than others. (*B*) Mean square displacement curves. Curves that fall below a straight line corresponding to the initial gradient (as these all do) represent constrained diffusive movement. Error bars indicate bootstrap-estimated SD. (*C*) Constrained diffusion rate (µm^2^/s) of proteins in the membrane. All tested proteins differ. (*D*) Constrained region length (µm) of proteins in the membrane. *P < 0.05, ***P < 0.01; ns, not significant.

We used TIRF-single particle tracking (SPT) to study the PM proteins p35S::paGFP-LTI6b, p35S::PIP2A-paGFP, pFLS2::FLS2-GFP, and pPIN3::PIN3-GFP as these cover a diverse range of functions from pathogen perception to morphogen transport and resource acquisition (Fig. 2 and Movie S1). TIRF-SP imaging and tracking can be performed with both photoactivatable GFP (paGFP) and GFP with overexpression or native promoters. However, expression needs to be not so bright as to saturate the detector. This was the case for GFP-linked protein expression driven by the PIN3 and FLS2 promoters in the *A. thaliana* hypocotyl. Diffusion rates (D) were calculated by fitting a constrained diffusion model to the initial 4 s of particle tracking data (Fig. 2). paGFP-LTI6b displayed a significantly greater diffusion rate (D = 0.063 ± 0.003 µm^2^/s, *P* < 0.01, Fig. 2*C* and *SI Appendix*, Fig. S2) compared with the other proteins. The aquaporin PIP2A-paGFP (D = 0.026 ± 0.004 µm^2^/s) displayed an enhanced diffusion rate compared with FLS2-GFP (D = 0.005 ± 0.004 µm^2^/s, *P* < 0.01) and PIN3-GFP (D = 0.012 ± 0.001 µm^2^/sec, *P* < 0.01, Fig. 2*C*). The FLS2-GFP diffusion rate was significantly lower than that of PIN3-GFP (*P* ≤ 0.05). Fitting a pure diffusion model to the first two points of each curve (instantaneous diffusion, D_i_) showed the same pattern for protein diffusion rates, demonstrating that our conclusions are robust to the choice of model (Fig. 2 and *SI Appendix*, Fig. S2). The constrained region length occupied by the diffusing particle was shown to be the same for PIP2A-paGFP, FLS2-GFP, and PIN3-GFP, with only paGFP-LTI6b showing a statistically significant increase in constrained region length size compared with the other proteins (*P* < 0.05–0.01, Fig. 2*D*). Thus, we have demonstrated by single particle tracking that PM proteins move at different speeds within the membrane even when areas that they move within are relatively similar in size.

### The Actin and Microtubule Cytoskeletons Differentially Regulate PM Protein Dynamics.

The cell surface exists as a continuum containing the cell wall, PM, and cytoskeleton (13). It had been shown by FRAP that incubation of seedlings with cytochalasin D or oryzalin which depolymerize actin microfilaments or microtubules, respectively, did not affect the dynamics of minimal membrane proteins (5). However, other work has demonstrated that SYMREM1 nanodomain formation is dependent on actin but not microtubule cytoskeletons (10), that depolymerization of microtubules results in larger punctae of REM1.2 in the membrane (19), and that HIR1 exists in microdomains and its dynamics are regulated by the actin and microtubule cytoskeletons (18). We, therefore, wanted to determine if cytoskeletal disruption affects nanodomain dynamics of other proteins.

**Fig. 3.**
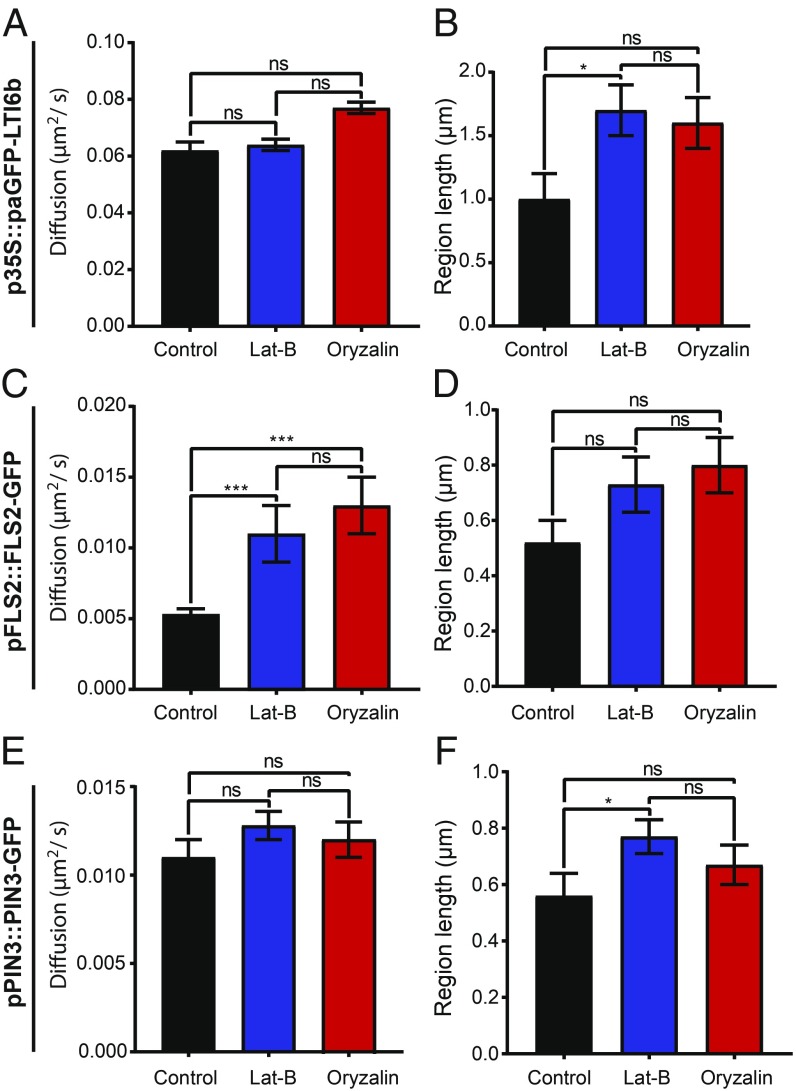
Actin cytoskeleton regulates the mobility of FLS2-GFP in the membrane. Plots show constrained diffusion rate (*A*, *C*, and *E*) and constrained region length (*B*, *D*, and *F*) of single particles within the PM of hypocotyl epidermal cells in controls and after treatment with latrunculin-B (1 h, 25 µM LatB) and oryzalin (1 h, 10 µM) to depolymerize the actin and microtubule cytoskeletons, respectively. (*A* and *B*) p35S::paGFP-LTI6b, (*C* and *D*) pFLS2::FLS2-GFP, and (*E* and *F*) pPIN3::PIN3-GFP. FLS2-GFP becomes significantly more dynamic when either cytoskeleton is depolymerized. *P < 0.05, ***P < 0.01; ns, not significant.

We confirmed that the concentrations of latrunculin-B (Lat-B) and oryzalin resulted in depolymerization of the actin and microtubule cytoskeletons (*SI Appendix*, Fig. S3). Using TIRF-SPT, upon actin or microtubule depolymerization, no changes were observed in the diffusion rate for PIN3-GFP and paGFP-LTI6b (Fig. 3 *A* and *E*, and Movie S2). Interestingly, both showed a significant increase in constrained region length after actin depolymerization (*P* < 0.05, Fig. 3 *B* and *F*). Conversely, upon actin or microtubule depolymerization, FLS2-GFP displayed an increase in diffusion rate (control, D = 0.0053 ± 0.0004 µm^2^/s; Lat-B, D = 0.011 ± 0.002 µm^2^/s; and oryzalin, D = 0.013 ± 0.002 µm^2^/s, *P* < 0.001, Fig. 3*C* and Movie S2), but not in constrained region length (Fig. 3*D*). This was also observed for instantaneous diffusion rates (*SI Appendix*, Fig. S4). Therefore, the actin and microtubule cytoskeletons can differentially regulate the mobility of proteins in the membrane.

### The Cell Wall Regulates PM Diffusion Rate, Region Length, and Nanocluster Size.

**Fig. 4.**
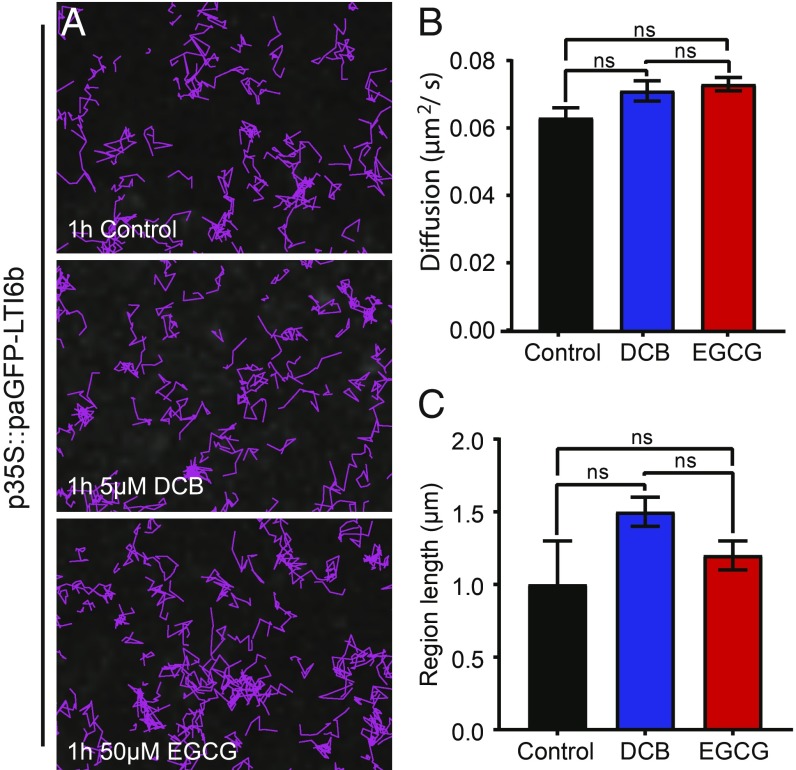
Single particle tracking reveals little effect of cell wall perturbation on paGFP-LTI6b dynamics. DCB was used to perturb cellulose synthesis and EGCG was used to perturb pectin methylation status of hypocotyl epidermal cells. (*A*) p35S::paGFP-LTI6b in control, and after 5 µM DCB and 50 µM EGCG treatments for 1 h each. Particles tracked over 60 s. (*B*) Constrained diffusion rate (µm^2^/s) of proteins in the membrane tracked over 4 s. (*C*) Constrained region length (µm) of proteins in the membrane during 4 s. ns, not significant.

**Fig. 5.**
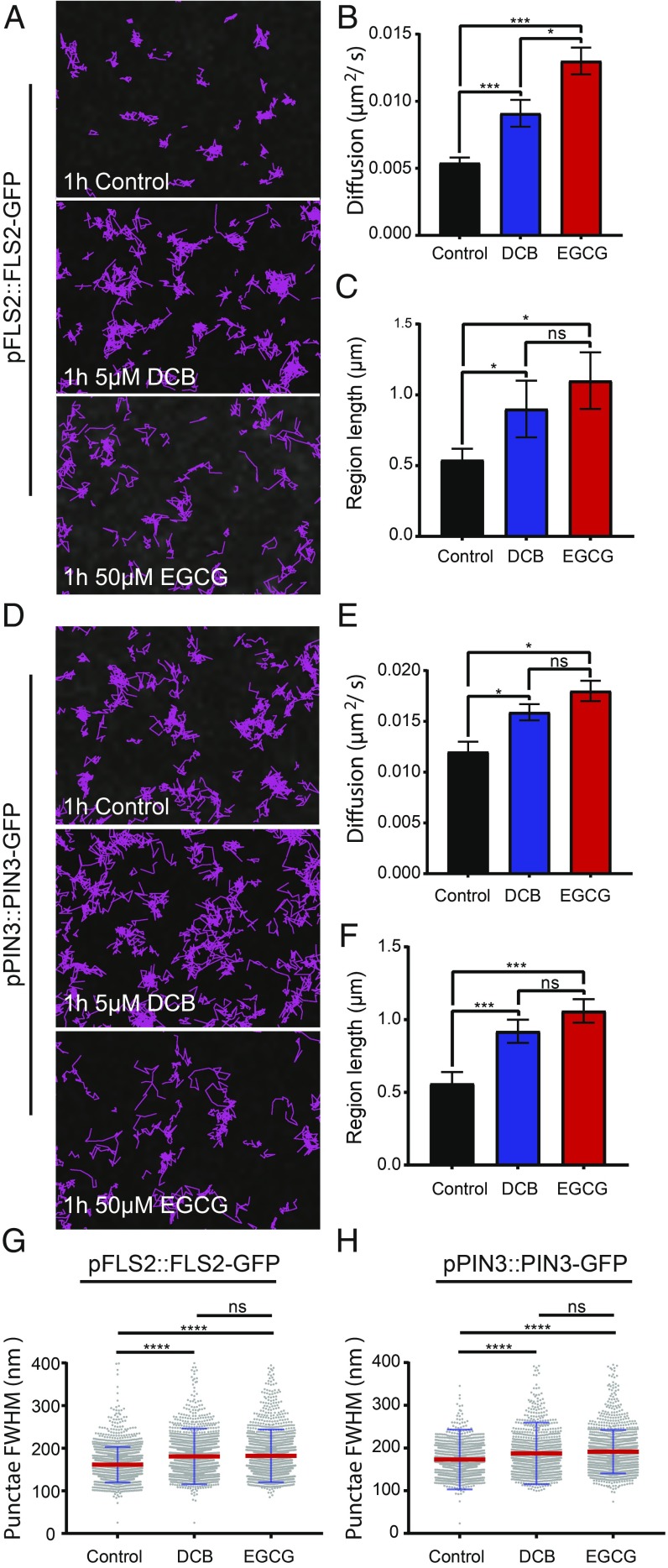
Cell wall perturbation alters diffusion rate, constrained region length, and cluster size of FLS2-GFP and PIN3-GFP. DCB was used to perturb cellulose synthesis and EGCG was used to perturb pectin methylation status of hypocotyl epidermal cells. (*A*–*C*) Nanodomain characteristics of pFLS2::FLS2-GFP and (*D*–*F*) pPIN3::PIN3-GFP in either controls, or after treatment with 5 µM DCB or 50 µM EGCG for 1 h. (*A* and *D*) Track length of single particles over 60 s. (*B* and *E*) Constrained diffusion rate over 4 s. (*C* and *F*) Constrained region length over 4 s. (*G* and *H*) FWHM measurement of cluster diameter. Scatter dot plot, red lines show mean value and blue error bars show SD. There was a significant increase or trend toward increase in all nanodomain characteristics for both proteins after cell wall perturbration. *P < 0.05, ***P < 0.01, ****P < 0.001; ns, not significant.

Previously our work has shown, using a combination of plasmolysis and protoplasting treatments that, upon removal of the cell wall constraint, protein lateral diffusion of minimal PM proteins with extracellular-facing GFP is increased (5). Therefore, in support and continuation of this work we hypothesized that the cell wall constrains the lateral diffusion rate of biologically functional proteins within the membrane. We performed TIRF-SPT imaging of paGFP-LTI6b, PIN3-GFP, and FLS2-GFP in combination with pharmacological perturbation of the cell wall (Figs. 4 and 5). PIN3-GFP and FLS2-GFP are both biologically active proteins with divergent function and were observed under control of their own promoters. The cellulose synthase-specific herbicide 2,6-dichlorobenzonitrile (DCB) (25) and epigallocatechin gallate (EGCG) which inhibits the native pectin methylesterase (26) were used to impair either cellulose synthesis or pectin methylation status (Figs. 4 and 5). Upon cell wall impairment with either, there was a nonsignificant trend toward increased diffusion rate (Fig. 4*B*) and constrained region length (Fig. 4*C*) for paGFP-LTI6b (Fig. 4 and Movie S3). There was, however, a significant increase in the instantaneous diffusion rate (D_i_) of paGFP-LTI6b upon cellulose or pectin perturbation (*SI Appendix*, Fig. S6 *A* and *B*, control, D_i_ = 0.066 ± 0.005 µm^2^/s; DCB, D_i_ = 0.085 ± 0.004 µm^2^/s; and EGCG, D_i_ = 0.085 ± 0.003 µm^2^/s). In addition, upon plasmolysis with either NaCl or mannitol, the paGFP-LTI6b diffusion rate was significantly increased in the PM (*SI Appendix*, Fig. S5 *A*–*E* and Movie S4). Therefore, minor cell wall perturbation by impairing individual components does not affect the constrained diffusion rate of paGFP-LTI6b, but separation of the cell wall from the PM by plasmolysis does.

We then studied the PM proteins PIN3-GFP and FLS2-GFP after cell wall perturbation (Fig. 5 and Movie S5). We chose PIN3-GFP and FLS2-GFP as their diffusion rates in untreated cells were low compared with paGFP-LTI6b and PIP2A-paGFP (Fig. 2). In addition, PIN3 is functionally active in the hypocotyl as the flow of auxin is constant throughout plant development. Conversely, FLS2 should not be signaling in the absence of its ligand, flg22 (27). Unlike paGFP-LTI6b, both PIN3-GFP and FLS2-GFP showed significantly increased diffusion rate and constrained region length upon treatment with either DCB or EGCG [FLS2-GFP control, D = 0.0054 ± 0.0004 µm^2^/s; DCB, D = 0.0091 ± 0.001 µm^2^/s; and EGCG, D = 0.013 ± 0.001 µm^2^/s, *P* < 0.001 (Fig. 5 *A*–*C*) and PIN3-GFP control, D = 0.012 ± 0.001 µm^2^/s; DCB, D = 0.0159 ± 0.0008 µm^2^/s; and EGCG, D = 0.018 ± 0.001 µm^2^/s, *P* < 0.05 (Fig. 5 *D*–*F*)]. Therefore, perturbation of either cellulose or pectin components of the cell wall results in these proteins diffusing faster and over a larger region length (Fig. 5). Furthermore, plasmolysis with either NaCl or mannitol caused an increase in diffusion rate and constrained region length for both (*SI Appendix*, Fig. S5 *F*–*O* and Movie S6), with the exception of the constrained region for FLS2-GFP (*SI Appendix*, Fig. S6*J*).

In combination with TIRF-SPT, Airyscan imaging of PIN3-GFP and FLS2-GFP demonstrated that nanodomain size significantly increases upon perturbation of either cellulose synthesis or pectin status (Fig. 5 *G* and *H*). FLS2-GFP nanodomain size, control, FWHM = 161.4 ± 41.5 nm; DCB, FWHM = 180.7 ± 65.35 nm; EGCG, FWHM = 182.1 ± 61.94 nm is shown in Fig. 5*G*. Nanodomain size after DCB and EGCG treatment was significantly greater than in controls (*P* ≤ 0.0001, ANOVA); however, there was no statistically significant difference between FLS2-GFP DCB- and EGCG-treated nanodomain size (*P* > 0.05, ANOVA). As with FLS2-GFP, PIN3-GFP nanodomain size was significantly greater after treatment with DCB or EGCG (*P* ≤ 0.0001, ANOVA), PIN3-GFP nanodomain size, control, FWHM = 173.1 ± 70.1 nm; DCB, FWHM = 187.6 ± 72.29 nm; EGCG; FWHM = 191.5 ± 50.92 nm (Fig. 5*H*). However, there was no significant difference between DCB- and EGCG-treated nanodomain size (*P* > 0.05, ANOVA). We also performed enzymatic degradation of the cell wall using cellulase and two different pectinase enzymes (*SI Appendix*, Fig. S7). These show that nanodomain size of both FLS2-GFP and PIN3-GFP is increased after enzymatic degradation of the cell wall, further supporting our observations with DCB and EGCG.

Therefore, for FLS2-GFP and PIN3-GFP upon either plasmolysis, or cellulose and pectin disruption, there is an increase in constrained diffusion rate, constrained region length, and nanodomain size. This demonstrates that the cell wall has a direct role in regulating both PIN3-GFP and FLS2-GFP protein dynamics and nanodomain size in the membrane.

## Discussion

### Proteins Reside in Different Sized Nanodomains and Display Different Dynamics in the Plasma Membrane.

We have shown that several plasma-membrane proteins form nanodomains which can be resolved with subdiffraction-limited imaging. The proteins we chose to image have diverse biological functions and have not been shown to have domains anchored into the cell wall as do, for example, FORMIN1 (17), AGP4 (5), or WAK1 and -2 (28). The auxin efflux transporter PIN2 has been shown using STED microscopy to form nanodomains of 100–200 nm in diameter which is the same observed by us for PIN3-GFP using Airyscan imaging (11). However, in the same investigation, BRI1 was found to have weak protein heterogeneity (11), which is in contradiction to our findings (*SI Appendix*, Fig. S1) and those of others (29). We have imaged hypocotyl epidermal cells while the BRI1 study was conducted using root epidermis. Tissue-specific differences such as the cell wall status, which we have shown to be important for nanodomain size (Fig. 5 and ref. 5), might explain these contradictory observations. We have shown that nanodomain size is significantly different for the various proteins investigated (Fig. 1 and *SI Appendix*, Fig. S1). Both FLS2 and BRI1 form nanodomains in the membrane (12, 29–31), which supports our study. However, the reported size for BRI1-GFP and FLS2-GFP nanodomains is significantly larger than we observe here (12). This is likely due to the imaging mode used and the image analysis methods employed.

Using TIRF-SPT, we have demonstrated that FLS2-GFP and PIN3-GFP have different diffusion rates within the plane of the PM. Furthermore, the dynamics of the proteins investigated are complex and not uniform. FLS2-GFP and PIN3-GFP have large extracellular domains (32, 33). Minimal membrane proteins which are PM anchored and have an intracellular GFP tag have faster diffusion rates than minimal membrane proteins which have extracellular GFP (2, 5). Therefore, the study of functional biologically relevant proteins which contain extracellular domains is more instructive than marker proteins without extracellular domains, although the dynamics of biologically functional PM-localized proteins which have no extracellular domains warrant investigation.

Protein diffusion rate differences exist in the plant PM for all proteins investigated in this study. This is similar to observations using dSTORM imaging of individual TCR molecules in activated human T cells (34) and proteins located in membrane sheets imaged with STED (35). Heterogeneity of membrane protein diffusion rates is a common theme across kingdoms. Note that all proteins imaged also form differently sized nanodomains within the PM. Heterogeneity of protein domain sizes and diffusion rates suggests that nanodomains of PM-localized proteins must show substantial crowding/overlap within the membrane. However, we have only imaged one labeled nanodomain at a time in this study. It will be interesting to extend this work to investigate protein species heterogeneity within the imaged nanodomains. Protein association within nanodomains would convey rapid functionality in multiprotein response pathways. Additionally, it could account for how signaling pathways which rely on common components such as FLS2 and BRI1 can lead to environmental or developmental responses as has been shown previously (12). This could also account for crosstalk between different pathways when components are localized to specific but partially overlapping nanodomains.

### The Actin and Microtubule Cytoskeleton Can Regulate the Diffusion of FLS2 but Not PIN3 and LTI6b.

We have demonstrated that the actin and microtubule cytoskeletons do not uniformly regulate the dynamics of PM proteins. The actin and microtubule cytoskeletons only regulate the diffusion rate of FLS2-GFP, which has increased lateral dynamics after depolymerization of either network (Fig. 3*C*). Both PIN3-GFP and paGFP-LIT6b showed no difference in diffusion rate upon cytoskeleton depolymerization, but did show an increase in the constrained region length when viewed as single particles (Fig. 3 *A*, *B*, *E*, and *F*). The constrained region length was not altered for FLS2-GFP by cytoskeleton depolymerization (Fig. 3). PIP2A has been shown previously by sptPALM imaging to have an increased diffusion rate upon depolymerization of the actin cytoskeleton (36) but no difference was reported for PIP2A upon oryzalin treatment to depolymerize the microtubule cytoskeleton. Actin and microtubule cytoskeleton regulation of some PM-localized proteins is further demonstrated by a recent report showing that the pathogen perception signaling protein BIK1 colocalizes to microtubules but not the actin cytoskeleton (12). In addition, actin or microtubule depolymerization resulted in loss of, and enlargement of nanodomain size of REM1.2, respectively (19). Furthermore, depolymerization of the actin, but not the microtubule cytoskeleton reduces nanodomain density of LYK3 (10). It has also been demonstrated that for HIR1, microtubules govern nanodomain dynamics within the PM, preferentially to actin microfilaments (18). Differential regulation of proteins by the cytoskeleton would contribute to proteins forming differently sized nanodomains and having differing diffusion rates in the membrane, which we and others have observed. All proteins investigated in this study show differently sized nanodomains with different dynamics in the membrane (Fig. 1 and *SI Appendix*, Fig. S1). The regulation of PM proteins by the cortical actin cytoskeleton has been investigated widely in mammalian cell systems and modeling has demonstrated that the actin cytoskeleton is sufficient to regulate heterogeneities in PM protein organization (37). This could partly account for the differences we observe in PM nanodomain size and dynamics in planta.

### The Cell Wall Regulates PM Nanodomain Size and Dynamics.

To determine effects that alterations in cell wall components might have on the diffusion rate of proteins within the PM, we perturbed cellulose synthesis and pectin methylation status. Neither of these treatments had a statistically significant effect on the constrained diffusion rate or region length of paGFP-LTI6b in the membrane (Fig. 4). Previous work (5) showed that treatment with isoxaben, an inhibitor of cellulose synthesis, results in more constrained movement of paGFP-LTI6b. Isoxaben and DCB have different modes of action and this could account for the discrepancy. Isoxaben results in removal of CESA complexes from the PM (14) and DCB stabilizes them (25).

PIN3-GFP and FLS2-GFP, however, showed changes in both diffusion rate and constrained region length upon cellulose (DCB induced) or pectin (EGCG induced) disruption (Fig. 5). Therefore, the cell wall acts to constrain the lateral mobility of these proteins within the PM. We have demonstrated that cell wall structure also regulates nanodomain size (Fig. 5 *G* and *H*). After 30 min of DCB-derived cell wall perturbation, cellulose synthase complexes are nonmotile in the PM (25) but no other significant changes are known to occur until much later, e.g., transcriptional changes, phytohormone induction, and lignin deposition occur at 4–7 h posttreatment (38). Therefore, minor cell wall perturbations rapidly affect PM nanodomain structure and dynamics. That such a short treatment has a profound effect on PM protein dynamics demonstrates how intimately related the cell wall and PM are. This could be a cellular mechanism that allows plant cells to rapidly respond to mechanical stimuli. It is interesting that separating the cell wall and PM as occurs during plasmolysis results in increased diffusion of paGFP-LTI6b, whereas specifically impairing a single cell wall component over a short time frame did not. This could be because the cell wall has a global effect on the dynamics of all proteins with the severity depending on the size of any extracellular domains or residues. In addition, a subset of proteins with extracellular residues such as PIN3-GFP and FLS2-GFP might chemically interact with cell wall domains as has been demonstrated for Formin1 (5), and breakage of these chemical bonds resulting from plasmolysis might destabilize the entire membrane structure. The dense extracellular matrix of brain synapses has been shown to regulate the lateral mobility of AMPA-type glutamate receptors (39). Therefore, the role of extracellular matrices in governing the dynamics of PM proteins is common across kingdoms.

It would be interesting to determine if changes in nanodomain size affect the signaling functions of either PIN3 or FLS2 and subsequent hormone transport or ligand binding. The pathogen receptor protein FLS2 has lowered lateral mobility when treated with flg22 in protoplasts (40). This has also been demonstrated for the aquaporin PIP2A which, upon salt stress, colocalizes with the membrane nanodomain marker FLOT1 and shows altered mobility within the membrane (41). LYK3, upon ligand binding and host cell infection shows reduced dynamics and increased stability in the membrane (10). In addition, nanodomains have been shown to be important for the activation of receptor-mediated signaling upon ligand perception and subsequent clathrin-mediated endocytosis (29). Therefore, cell wall regulation of PM nanodomains can be interpreted as of fundamental importance to signaling in planta.

To conclude, we have shown that a number of PM proteins form nanodomains within the PM and that these are resolvable using subdiffraction-limited techniques such as the Zeiss Airyscan system. These nanodomains are of different sizes and their dynamics and size can be differentially regulated by the actin and microtubule cytoskeletons and the cell wall. As yet, very limited information exists as to how PM proteins form nanodomains. We demonstrate here that the cell wall plays a key role in regulation of protein nanodomain size and lateral mobility for the pathogen receptor FLS2 and the auxin transporter PIN3. We hypothesize that the cytoskeleton and cell wall slow nanodomain dynamics sufficiently to allow relatively static distribution of functional proteins so that they are well placed spatially for optimum association.

## Materials and Methods

For full and detailed methods please see *SI Appendix*, *Materials and Methods*.

### Plant Material.

*A. thaliana* hypocotyl cells from 5-d-old seedlings were imaged. Plants were grown vertically on 0.5× strength MS plates. Chemical treatments were performed for 1 h. All drugs used were stored as 1,000× stocks and diluted in MS liquid media.

### Live Cell Imaging and Analysis.

Seedlings were imaged with no. 1.5 coverslips stuck down with double-sided tape. For Airyscan and confocal imaging a Zeiss 880 system was used. FWHM was determined using the FIJI implementation of ImageJ, with statistics and graphs produced in Graphpad Prism v7. TIRF imaging was performed as described previously (5). TIRF-SPT was performed as described previously (42) with some modifications.

## Supplementary Material

Supplementary File

Supplementary File

Supplementary File

Supplementary File

Supplementary File

Supplementary File

Supplementary File
