# Supplementary material for: The cell wall regulates dynamics and size of plasma-membrane nanodomains in Arabidopsis
Source: Proc Natl Acad Sci U S A. 2019 Jun 10;116(26):12857–62. doi: 10.1073/pnas.1819077116 (PMC6601011; doi:10.1073/pnas.1819077116)
Supplement: Supplementary File [file pnas.1819077116.sapp.pdf]

PNAS
www.pnas.org

Supplementary Information for

The cell wall regulates dynamics and size of plasma-membrane nanodomains in *Arabidopsis*.

McKenna JF[1], Rolfe DJ[2], Webb SED[2,3], Tolmie AF[1], Botchway SW[2], Martin-Fernandez ML[2], Hawes C[1] & Runions J[1].

Corresponding author: Professor John Runions
Email: jrunions@brookes.ac.uk

This PDF file includes:

Supplementary Materials and Methods
Figs. S1 to S7
Tables S1 to S2
Captions for movies S1 to S6
References for SI reference citations

Other supplementary materials for this manuscript include the following:

Movies S1 to S6

www.pnas.org/cgi/doi/10.1073/pnas.1819077116

Supplementary Information Text

Supplementary Information Materials and Methods

Plant material

The *Arabidopsis thaliana* seed lines used have been previously described; p35S::paGFP-LTI6b (1), pFLS2::FLS2-GFP (2), pPIN3::PIN3-GFP (3), p35S::PIP2A-GFP (4), p35S::PIP2A-paGFP (5), pREM1.3::YFP-REM1.3 (6), pBRI1::BRI1-GFP (7), p35S::GFP-TUA (8) and p35S::GFP-fABD2 (9). Seeds were surface sterilized in 70% ethanol for 5 minutes, 50% bleach for 5 minutes and washed four times with water. Seeds were placed on square agar plates composed of ½ strength MS with MES and 0.8% Phytagel, pH5.7. Seedlings were then stratified on plates for 2 days at 4°C in the dark and then placed into a growth chamber set to 16:8h long days, 23°C, and 120µEinstein's for 5 days before imaging. Hypocotyl cells from 5 day old seedlings were imaged for all experiments.

Chemical treatments

A. thaliana seedlings were treated in 8ml dH_2O in 6 well plates for 1 hour with the following concentrations, all made from 1000X stocks; 5µM DCB, 50µM EGCG, 0.5M mannitol, 100mM NaCl, 25µM latrunculin-B and 10µM oryzalin. DCB, latrunculin-B and oryzalin stocks were dissolved in DMSO and the EGCG stock was dissolved in ethanol. For enzymatic degradation of the cell wall Cellulase (Onozuka R-10, Serva catalogue number 16419), Pectinase (Sigma catalogue number P2611) and Macerozyme R-10 (Serva catalogue number 28302) were dissolved in dH_2O (pH 5.7) to a concentration of 1%.

Confocal microscopy

Seedlings were imaged after five days of growth by mounting them in dH_2O on microscope slides with no1.5 coverslips. Slides and coverslips were held down with micropore tape. A Zeiss LSM880 equipped with an Airyscan detector was used. Airyscan imaging was performed using 488 and 514nm excitation for GFP and YFP, respectively. Lasers were used at 1% transmission and a dual 495-550 band pass filter and 570nm long pass filter.

For standard confocal imaging the same emission wavelength was imaged with a GaAsP detector. To avoid chlorophyll autofluorescence a 615nm shortpass filter was used. A 100x/1.46 DIC M27 Elyra oil immersion lens was used for all imaging. A 5X zoom was used to image flat membrane sheets and imaging conditions were all set according to Zeiss optimal Airyscan framesize (for 5X zoom, 404x404). Frame sizes were kept the same for standard confocal imaging. For single particle experiments, sample size (n) = a minimum of 12 cells imaged across 3 biological replicates per condition, the number of single particles tracked per condition is displayed in Table S1. For all Airyscan data n = ≤64 punctae were measured/cell for 36 cells across three biological repeats, exact numbers for each condition can be seen in Table S2.

Airyscan image analysis

PM protein nanodomain size was determined by imaging using the above conditions. Using the FIJI implementation of ImageJ, an 8X8 grid was placed over the image and line profiles determined for the brightest nanodomain in each grid cell. The full width half maximum (FWHM) of these line profiles was then determined and this data was collated in Graphpad Prism version 7. Scatter dot plots were produced with error bars denoting the standard deviation. ANOVA with multiple comparisons was used to assess nanodomain size differences for different proteins. Kymographs were produced from 55 subsequent images comprising 8 seconds of imaging the PM. The Multiline kymograph plugin in FIJI was used to produce a kymograph with the line originating in the bottom left corner at a 45 degree angle to the top right for each data-set.

TIRF-SP Imaging

TIRF imaging was performed as described in (1) using an inverted microscope (Axio Observer, Zeiss) equipped with a 100X objective (α-Plan-Apochromat, NA = 1.46; Zeiss) and TIRF slider (Zeiss), 488-nm laser excitation (Stradus Versalase, Vortran), HQ525/50-nm emission filter (Chroma), and an electron-multiplication CCD (iXon+; Andor). The exposure time was 50 ms.

TIRF-SPT - Particle tracking

Single-molecule time series data were analysed using the multidimensional analysis software described previously (10). Briefly, this software performs frame-by-frame Bayesian

segmentation to detect and measure features to sub-pixel precision, then links these features through time to create tracks using a simple proximity-based algorithm. The software determines cubic polynomial registration transformations from images of fluorescent beads. Feature detection and tracking was performed independently in each channel. Intracellular regions of the field of view suitable for the single-molecule analysis -
excluding dense emission from the cell membrane - were manually segmented and only particles detected in these regions were included in subsequent analysis.

From single particle tracks, mean squared displacement (MSD) curves were calculated as: $\mathrm{MSD}(\Delta T)=\langle|\mathbf{r}_i(T+\Delta T)-\mathbf{r}_i(T)|^2\rangle$ where $|\mathbf{r}_i(T+\Delta T)-\mathbf{r}_i(T)|$ is the displacement between position of track *i* at time *T* and time *T*+*ΔT* and the average is over all pairs of points separated by *ΔT* in each track. The errors in the MSD curve were calculated by repeating the MSD curve calculation 200 times, each time on a different synthetic dataset created by randomly resampling with replacement the tracks present within each dataset (bootstrap resampling (11)). The distribution of $\mathrm{MSDboot}_j(\Delta T)$ curves about the MSD curve for the unresampled data, MSD(*ΔT*), should be close to the distribution of MSD(*ΔT*) about the true MSD curve (11). Therefore a posterior sample of 200 MSD curves $\mathrm{MSDpost}_j(\Delta T)$ was calculated from these 200 bootstrap MSD curves $\mathrm{MSDboot}_j(\Delta T)$ (j=1..200):

$$MSD(\Delta T) - MSDpost_j(\Delta T) = MSDboot_j(\Delta T) - MSD(\Delta T)$$

so

$$MSDpost_j(\Delta T) = 2MSD(\Delta T) - MSDboot_j(\Delta T)$$

Subsequent model fits (see below) were performed on each posterior MSD curve sample to naturally yield joint posterior samples of the fitted model parameters suitable for determining confidence intervals, error bars and statistical tests. A χ^2 fit was performed for each posterior sample using the standard deviation of the posterior MSDs at each ΔT as the error estimate for calculating χ^2.

The first model fitted was free diffusion with parameters diffusion rate (D) and localisation error (σ_{loc}), which was fitted to the first two points on the curve, for which:

$MSD(\Delta T) = 4D\Delta T + 4\sigma_{loc}^2$ (12)

The second model fitted was confined diffusion. This models the particles as undergoing Brownian diffusion but only within a limited 2-dimensional area of side length L from which they cannot escape during the observation period. If the diffusion rate is D, and localisation error is σ_{loc} *then the model MSD curve is:*

$MSD(\Delta T) = \frac{L^2}{3}\left[1 - exp\left(\frac{-12D\Delta T}{L^2}\right)\right] + 4\sigma_{loc}^2$ (12)

The confidence intervals for each parameter were chosen as the midpoint ± half width of shortest interval containing 69% of the posterior probability for that parameter.

We assume for the null hypothesis that posterior samples 1 and 2 correspond to the same value of quantity x, the probability of a given difference Δx is the same as the measured probability of Δx about its mean, i.e.

$$P(\Delta x|NULL) = P(\Delta x - \langle \Delta x \rangle | sample1, sample2)$$

The probability that $|\Delta x|$ is at least $\langle \Delta x \rangle$ given the null hypothesis is then:

$$P(|\Delta x| > |\langle \Delta x \rangle|| NULL) = \int_{|\langle \Delta x \rangle|}^{\infty} P\,(|\Delta x - \langle \Delta x \rangle|| sample1, sample2)$$

We used this as a non-parametric p-value for the null hypothesis that the two posterior samples measure the same value. In the case of normally distributed posteriors from normally distributed sample measurements this gives the same p-values as the 2-sided Welch’s t-test.

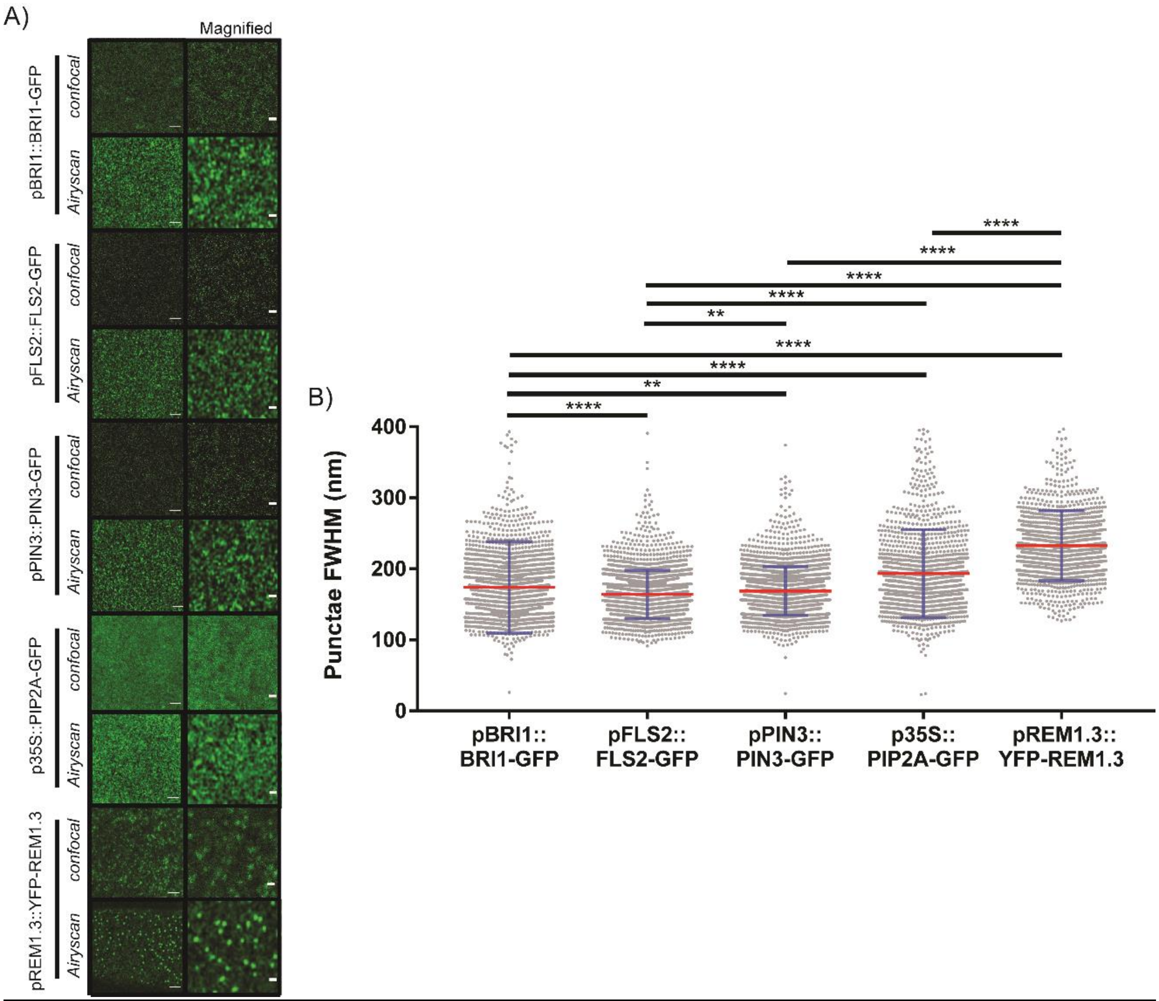

Fig. S1. Comparison of Confocal and Airyscan imaging of PM nanodomains. A) Comparison of Confocal and Airyscan imaging of a number of PM localised proteins, scale bar denotes 500nm. B) Scatter plot showing nanodomain size calculated using FHWM of line profiles. FLS2-GFP, PIN3-GFP and YFP-REM1.3 also shown in Fig. 1. ** = $p \leq 0.01$, **** = $p \leq 0.0001$ ANOVA. Red line denotes mean, blue error bars denote standard deviation. Please note, FLS2, PIN3 and REM1.3 data also in figure 1 but shown here for complete comparison. Number of nanodomain quantified = BRI1; 2301, FLS2; 2245, PIN3; 2235, PIP2A; 1308, REM1.3; 1420 collected from 3 experimental repeats.

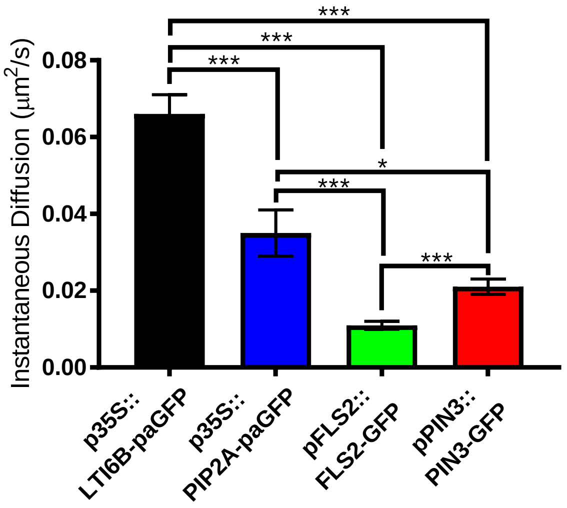

Fig. S2. . Instantaneous diffusion values for TIRF single particle tracking of PM proteins. Instantaneous diffusion rates of p35S::paGFP-LTI6b, p35S::PIP2A-paGFP, pFLS2::FLS2-GFP and pPIN3::PIN3-GFP PM localised proteins determined by TIRF-SPT. *=p<0.05, ***=p<0.01.

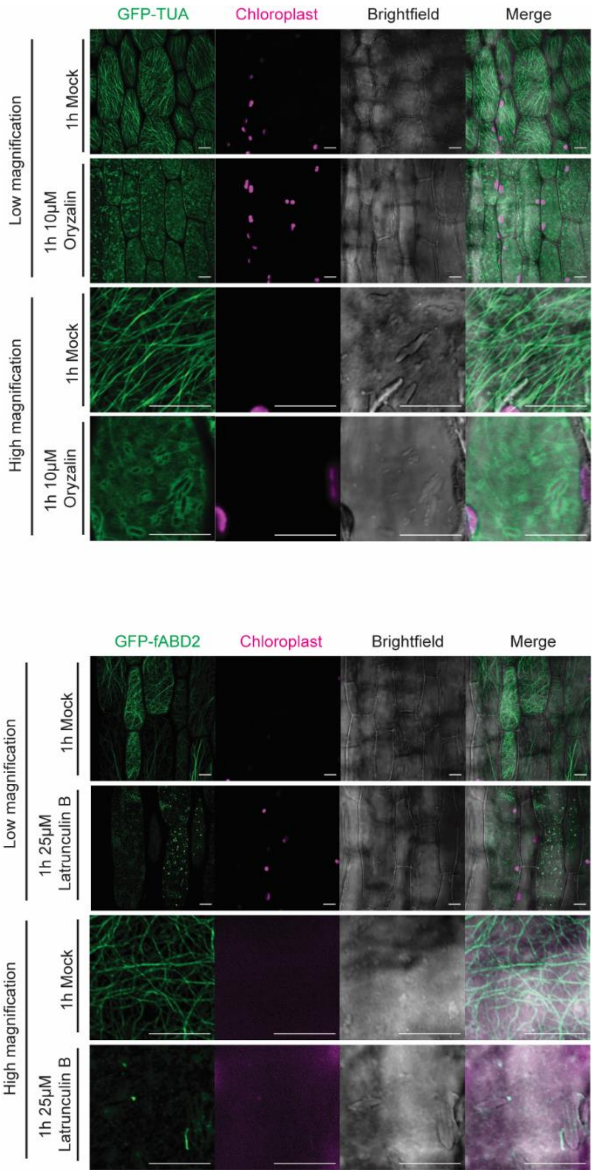

Fig. S3. The Latrunculin B and Oryzalin concentrations used depolymerize actin and microtubules respectively. The treatments performed for actin cytoskeleton depolymerization (1h 25µM Latrunculin B) and microtubule depolymerization (1h 10µM Oryzalin) are working as expected. GFP-fABD2 and GFP-TUA transgenic lines were used to label actin and microtubule cytoskeletons respectively. 63x lens used, 1X and 5X digital magnification is provided. Scale bars denote 10µm. 12 cells imaged across three seedlings. Representative images shown.

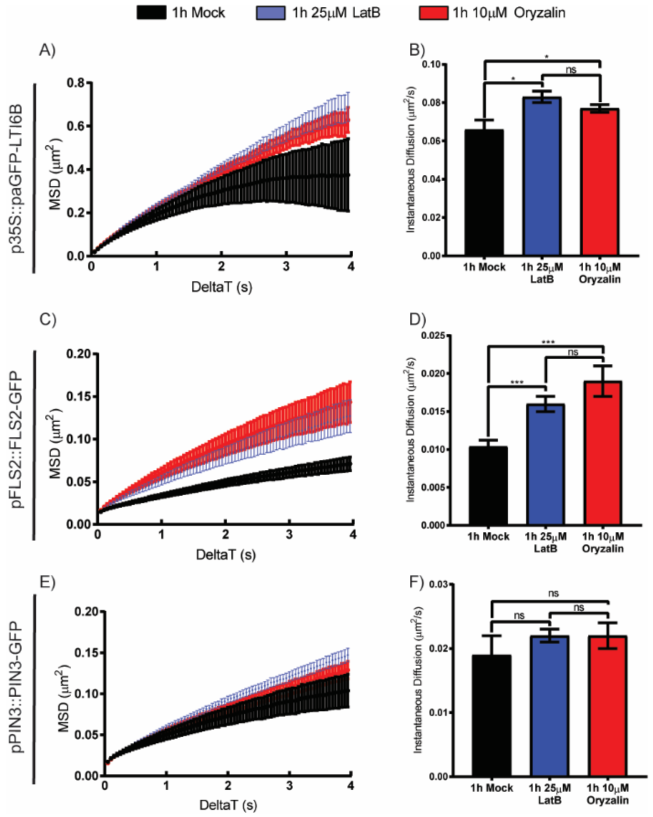

Fig. S4. Instantaneous diffusion values for TIRF single particle tracking of p35S:: paGFP-LTI6b, pPIN3::PIN3-GFP and pFLS2::FLS2-GFP during cytoskeleton perturbation. Instantaneous diffusion rates determined by TIRF-SPT during actin (Lat-B) and microtubule (oryzalin) cytoskeleton perturbation. *** = p<0.01, *=p<0.05, ns=not significant.

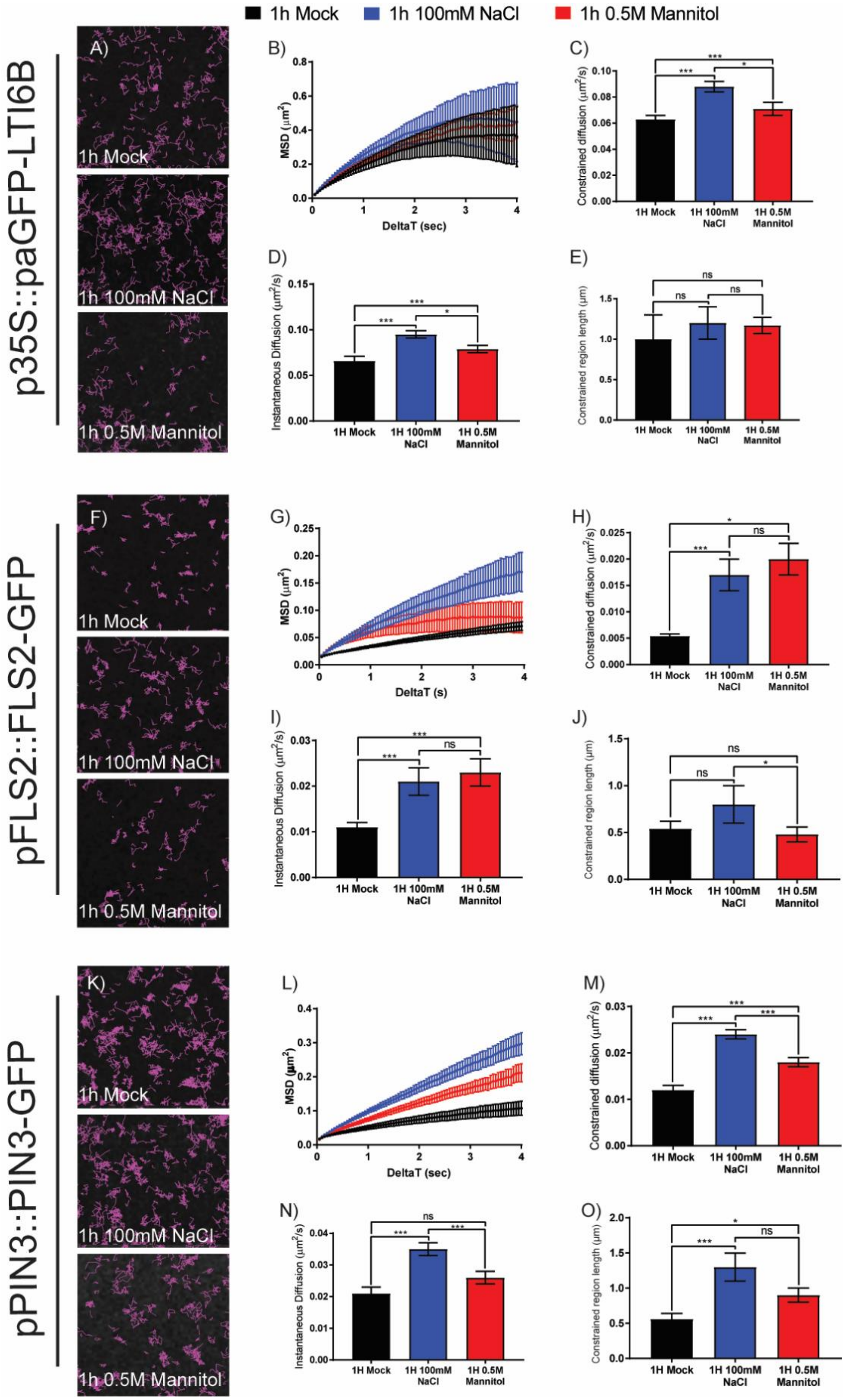

Fig. S5. Plasmolysis causes changes in single particle dynamics for p35S::paGFP-LTI6b, pFLS2::FLS2-GFP and pPIN3::PIN3-GFP. A) p35S::paGFP-LTI6B single

particle tracks in mock, 100mM NaCl and 0.5M Mannitol plasmolysis. B) MSD curve of p35S::paGFP-LTI6B plasmolysis treatments. C) Instantaneous diffusion rates of p35S::paGFP-LTI6B single particles tracked during plasmolysis treatments. D) Constrained diffusion rates of p35S::paGFP-LTI6B particles tracked over 4s during plasmolysis treatments. E) Constrained area of p35S::paGFP-LTI6B particles tracked over 4 seconds during plasmolysis treatments. F) pFLS2::FLS2-GFP single particle tracks in mock, 100mM NaCl and 0.5M Mannitol plasmolysis. G) MSD curve of pFLS2::FLS2-GFP plasmolysis treatments. H) Instantaneous diffusion rates of pFLS2::FLS2-GFP single particles tracked during plasmolysis treatments. I) Constrained diffusion rates of pFLS2::FLS2-GFP particles tracked over 4 seconds during plasmolysis treatments. J) Constrained area of pFLS2::FLS2-GFP particles tracked over 4 seconds during plasmolysis treatments. K) pPIN3::PIN3-GFP single particle tracks in mock, 100mM NaCl and 0.5M Mannitol plasmolysis. L) MSD curve of pPIN3::PIN3-GFP plasmolysis treatments. M) Instantaneous diffusion rates of pPIN3::PIN3-GFP single particles tracked during plasmolysis treatments. N) Constrained diffusion rates of pPIN3::PIN3-GFP particles tracked over 4 seconds during plasmolysis treatments. O) Constrained area of pPIN3::PIN3-GFP particles tracked over 4 seconds during plasmolysis treatments. ns=not significant, *=$p<0.05$, *** $p<0.01$.

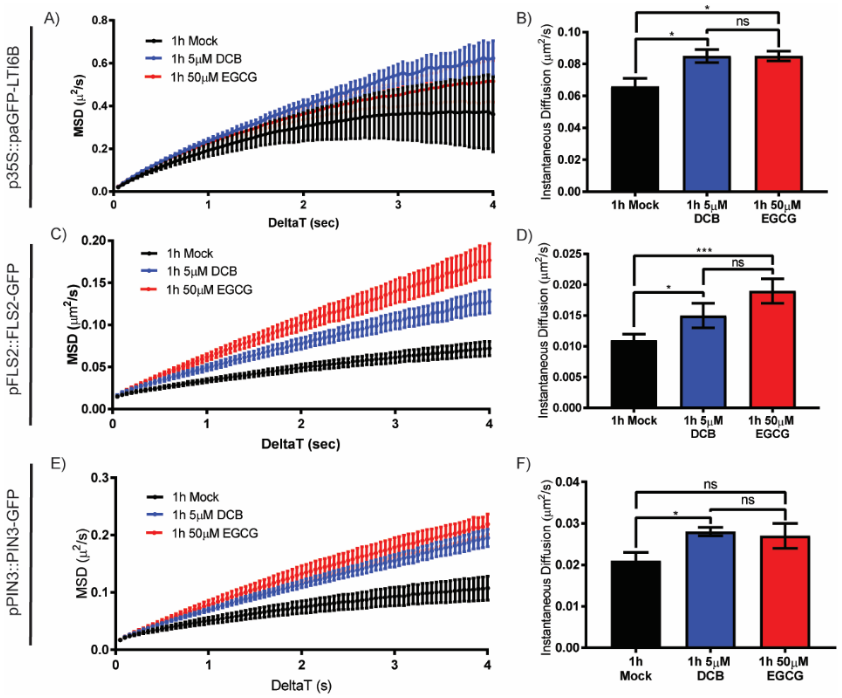

Fig. S6. Instantaneous diffusion values for p35S::paGFP-LTI6b, pPIN3::PIN3-GFP and pFLS2::FLS2-GFP during cell wall perturbation. Instantaneous diffusion rates of p35S::paGFP-LTI6B, pFLS2::FLS2-GFP and pPIN3::PIN3-GFP with mock, 5µM DCB and 50µM EGCG treatment. ns=not significant, *=p<0.05.

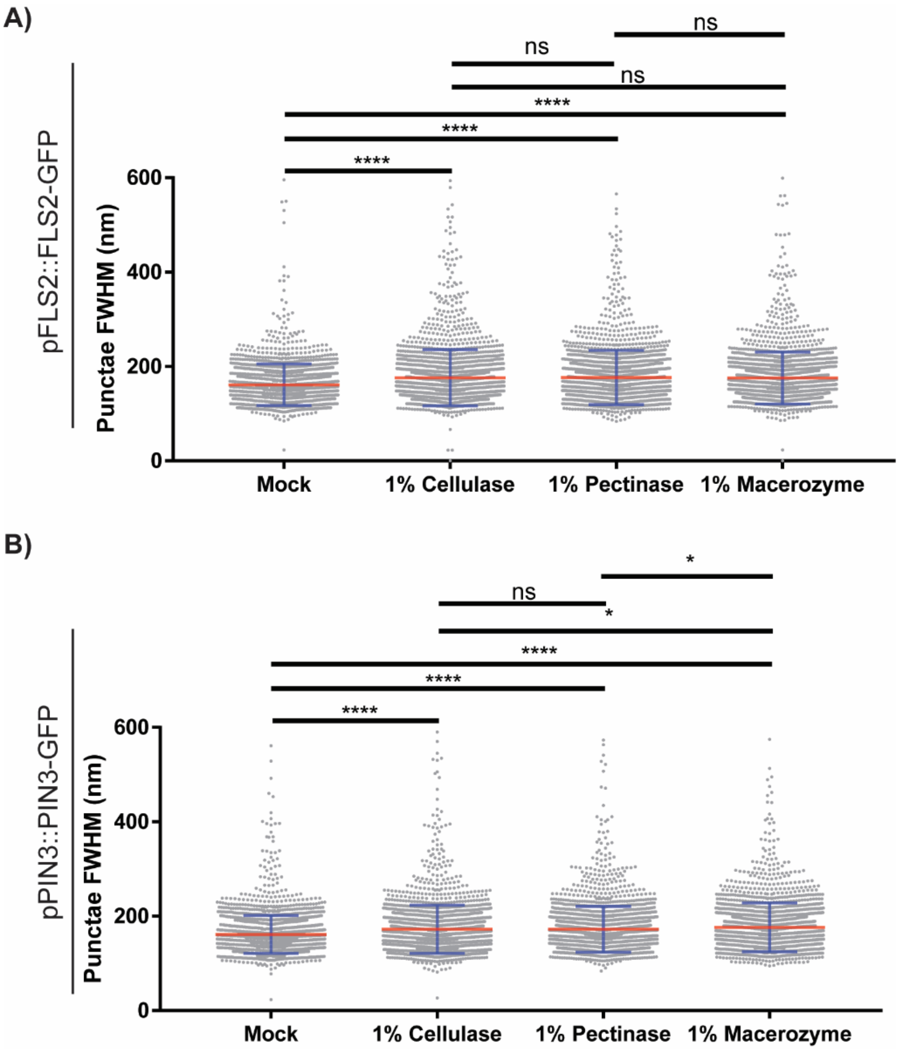

Fig. S7. Enzymatic degradation of Cellulose or Pectin of the cell wall results in increased nanodomain size in 5 day old *Arabidopsis* hypocotyl cells. A) FLS2-GFP and B) PIN3-GFP nanodomain size was determined using Airyscan imaging and FWHM measurements. Treatments were performed for 1 hour with Cellulase to digest cellulose, Pectinase to digest pectin and Macerozyme which digests hemicellulose and pectin components of the cell wall. Enzymatic degradation of the cell wall by all treatments resulted in increased nanodomain size. FLS2-GFP 1h mock FWHM = 168 ± 44.54nm, Cellulase; FWHM = 175.8nm ± 59.89nm, Pectinase; FWHM = 176.3 ± 57.81 and Macerozyme; FWHM = 157.4 ± 55,54 nm. PIN3-GFP 1h mock FWHM = 161.4 ± 40.18nm, Cellulase; FWHM = 171.7 ± 51.8nm, Pectinase; FWHM = 172 ± 48.66nm and Macerozyme; FWHM = 176.1 ± 52.1nm. ANOVA statistical test performed; ns = $p \geq 0.05$, * = $p \leq 0.05$ and **** = $p \leq 0.0001$. N = ≥2234 nanodomains from 36 cells across 12 seedlings from 3 experimental repeats.

	p35S::paGFP-LTI6B	pFLS2::FLS2-GFP	pPIN3::PIN3-GFP	p35S::PIP2A-paGFP
**Mock**	2799	2617	5059	8500
**5µM DCB**	2173	2315	5749	N/A
**50µM EGCG**	3692	2724	5562	N/A
**20µM Isoxaben**	4819	2117	7214	N/A
**100mM NaCl**	2130	2006	2055	N/A
**0.5M Mannitol**	1249	1364	2078	N/A
**25µM Latrunclin B**	5062	2781	4959	N/A
**10µM Oryzalin**	2325	3400	4183	N/A

Table S1. Number of tracks analysed per construct per treatment for single particle imaging.

**Genotype**	**Condition**	**Experiment**	**# measured**
pBRI1::BRI1-GFP	Mock	nanodomain comparison	2301
pFLS2::FLS2-GFP	Mock	nanodomain comparison	2245
pPIN3::PIN3-GFP	Mock	nanodomain comparison	2235
p35S::PIP2A-GFP	Mock	nanodomain comparison	1308
pREM1.3::YFP-REM1.3	Mock	nanodomain comparison	1420
pPIN3::PIN3-GFP	Mock	Cell wall perturbation	1564
pPIN3::PIN3-GFP	5µM DCB	Cell wall perturbation	1234
pPIN3::PIN3-GFP	50µM EGCG	Cell wall perturbation	1470
pFLS2::FLS2-GFP	Mock	Cell wall perturbation	2164
pFLS2::FLS2-GFP	5µM DCB	Cell wall perturbation	2249
pFLS2::FLS2-GFP	50µM EGCG	Cell wall perturbation	2207
pFLS2::FLS2-GFP	1h Mock for cell wall enzymatic degradation	Cell wall enzymatic degradation	2388
pFLS2::FLS2-GFP	1h 1% Cellulase	Cell wall enzymatic degradation	2306
pFLS2::FLS2-GFP	1h 1% Pectinase	Cell wall enzymatic degradation	2276
pFLS2::FLS2-GFP	1h 1% Macerozyme	Cell wall enzymatic degradation	2234
pPIN3::PIN3-GFP	1h Mock for cell wall enzymatic degradation	Cell wall enzymatic degradation	2557
pPIN3::PIN3-GFP	1h 1% Cellulase	Cell wall enzymatic degradation	2308
pPIN3::PIN3-GFP	1h 1% Pectinase	Cell wall enzymatic degradation	2360
pPIN3::PIN3-GFP	1h 1% Macerozyme	Cell wall enzymatic degradation	2311

Table S2. Number of nanodomain size measurements per construct per treatment for Airyscan imaging. Nanodomain comparison data used in figures 1 and S1. Cell wall perturbation used in Figure 5.

Movie S1. Type or paste caption here.

Movie S1. TIRF single particle tracking of paGFP-LTI6b, PIP2A-paGFP, FLS2-GFP and PIN3-GFP shows they diffuse at different rates and occupy differing sized areas within the PM.

Movie S2. TIRF single particle tracking of paGFP-LTI6B, PIP2A-paGFP, FLS2-GFP and PIN3-GFP during control, actin (Lat-B) and microtubule (Oryzalin) depolymerisation.

Movie S3. TIRF single particle tracking of paGFP-LTI6b in the PM during cell wall perturbation.

Movie S4. TIRF single particle tracking of paGFP-LTI6b in the PM during plasmolysis.

Movie S5. TIRF single particle tracking of FLS2-GFP and PIN3-GFP during cell wall perturbation.

Movie S6. TIRF single particle tracking of FLS2-GFP and PIN3-GFP during plasmolysis.

References

1. Martinière A, et al. (2012) Cell wall constrains lateral diffusion of plant plasma-membrane proteins. *Proc Natl Acad Sci U S A* 109(31):12805–10.
2. Robatzek S, et al. (2006) Ligand-induced endocytosis of the pattern recognition receptor FLS2 in Arabidopsis service Ligand-induced endocytosis of the pattern recognition receptor FLS2 in Arabidopsis. 537–542.
3. Zádníková P, et al. (2010) Role of PIN-mediated auxin efflux in apical hook development of Arabidopsis thaliana. *Development* 137(4):607–17.
4. Boursiac Y, et al. (2005) Early Effects of Salinity on Water Transport in Arabidopsis Roots . Molecular and Cellular Features of Aquaporin Expression 1. 139(October):790–805.
5. Martinière A, et al. (2011) Homeostasis of plasma membrane viscosity in fluctuating temperatures. *New Phytol* 192(2):328–37.
6. Jarsch IK, et al. (2014) Plasma Membranes Are Subcompartmentalized into a Plethora of Coexisting and Diverse Microdomains in Arabidopsis and Nicotiana benthamiana. *Plant Cell* 26(4):1698–1711.
7. Di Rubbo S, et al. (2013) The clathrin adaptor complex AP-2 mediates endocytosis of brassinosteroid insensitive1 in Arabidopsis. *Plant Cell* 25(8):2986–97.

8. Ueda K, Matsuyama T, Hashimoto T (1999) Visualization of microtubules in living cells of transgenic Arabidopsis thaliana Rapid communication. *Protoplasma* (206):201–206.
9. Sheahan MB, Staiger CJ, Rose RJ, McCurdy DW (2004) A green fluorescent protein fusion to actin-binding domain 2 of Arabidopsis fimbrin highlights new features of a dynamic actin cytoskeleton in live plant cells. *Plant Physiol* 136(4):3968–3978.
10. Rolfe DJ, et al. (2011) Automated multidimensional single molecule fluorescence microscopy feature detection and tracking. *Eur Biophys J* 40(10):1167–86.
11. Press WH, Teukolsky SA, Vetterling WT, Flannery BP (1992) *Numerical Recipes in C: The Art of Scientific Computing, Volume 1* (University of Cambridge).
12. Bruchle C, Lamb DC, Michaelis J eds. (2009) *Single Particle Tracking and Single Molecule Energy Transfer* (Wiley-VCH Verlag GmbH & Co. KGaA, Weinheim, Germany) doi:10.1002/9783527628360.
